# Discrimination of natural acoustic variation in vocal signals

**DOI:** 10.1038/s41598-020-79641-z

**Published:** 2021-01-13

**Authors:** Adam R. Fishbein, Nora H. Prior, Jane A. Brown, Gregory F. Ball, Robert J. Dooling

**Affiliations:** 1 Department of Psychology, University of Maryland, Biology-Psychology Bldg., 4094 Campus Dr., College Park, MD 20742 USA; 2 Neuroscience and Cognitive Science Program, University of Maryland, College Park, MD USA

**Keywords:** Animal behaviour, Auditory system, Social behaviour, Social neuroscience

## Abstract

Studies of acoustic communication often focus on the categories and units of vocalizations, but subtle variation also occurs in how these signals are uttered. In human speech, it is not only phonemes and words that carry information but also the timbre, intonation, and stress of how speech sounds are delivered (often referred to as “paralinguistic content”). In non-human animals, variation across utterances of vocal signals also carries behaviorally relevant information across taxa. However, the discriminability of these cues has been rarely tested in a psychophysical paradigm. Here, we focus on acoustic communication in the zebra finch (*Taeniopygia guttata*), a songbird species in which the male produces a single stereotyped motif repeatedly in song bouts. These motif renditions, like the song repetitions of many birds, sound very similar to the casual human listener. In this study, we show that zebra finches can easily discriminate between the renditions, even at the level of single song syllables, much as humans can discriminate renditions of speech sounds. These results support the notion that sensitivity to fine acoustic details may be a primary channel of information in zebra finch song, as well as a shared, foundational property of vocal communication systems across species.

## Introduction

The parallels between acoustic communication in humans and non-human animals have fascinated casual observers as well as scientific researchers at least as far back as Aristotle^1,2^. This has motivated widespread investigation into how complex “information” and “meaning” are encoded in vocal signals^3–6^. These studies in both humans and non-human animals (hereafter “animals”) have been dominated by a search for linguistic content: identifying basic units and categories of vocalizations (such as words in humans and call types in non-human primates) and describing how these units may be combined into meaningful sequences^7–13^. But comparing the linguistic capabilities of humans and animals is difficult because these processes are largely internal, and animals appear to lack anything comparable to human semantics and syntax^14–18^.

In contrast to the linguistic domain, both humans and animals can communicate complex information through subtle variation in acoustic features of the “voice” (e.g. pitch, intensity, timbre, and intonation)^19–21^. Between individuals, this acoustic variation can encode information such as individual and group identity^22–27^. Within individuals, this acoustic variation can carry socially-relevant information such as emotional or motivational state^28–35^. For example, humans can perceptually categorize at least eight, and perhaps more than twelve, affective states from variation in the voice alone^36,37^. This shared ability of humans and animals to extract information from such fine acoustic variation is likely an important shared biological foundation of acoustic communication^38^.

Among acoustic communication systems, birdsong has long been a dominant model of human speech and vocal learning, and considerable research has been dedicated to describing the significance of variation in rhythm, timing, and the number and order of elements in birdsong within and across species^2,39–41^. Birdsong also exhibits subtle acoustic variation across renditions and contexts (e.g.^42–44^), but the significance of this information in communication has until recently gone largely unexplored. We now know that birds are acutely sensitive to variation in acoustic fine structure—the rapid fluctuations in amplitude and frequency within the envelope of a sound waveform^45–47^. Studies with natural song and calls confirm that songbirds use the acoustic structure of vocal signals for individual recognition^25,27^. Additionally, songbirds are exquisitely sensitive to manipulations of individual syllables, while some species, at least, are relatively insensitive to changes in syllable order^27,48^, suggesting that fine acoustic variation, such as across renditions of song syllables, may be a primary carrier of information.

In the current study, we used a psychophysical paradigm to ask whether birds can perceive naturally occurring variation in their vocal signals that are typically judged to be the same by conventional spectrographic analyses. The zebra finch (*Taeniopygia guttata*) is particularly well suited for asking this question since males learn a single highly stereotyped motif, comprised of 3–8 harmonic syllables in a fixed sequence, which they repeat multiple times in a song bout^49^. Here, we tested how well zebra finches can discriminate different renditions of song syllables from a given male. As a check on whether this ability reflects a general or specialized perceptual process, we also tested zebra finches on a task involving discriminating different renditions of human vowels from the same talker. Finally, for comparison, we tested the ability of human participants to discriminate renditions of zebra finch motifs and human vowels.

## Results

### Zebra finches can discriminate renditions of song motifs and syllables

**Figure 1:**
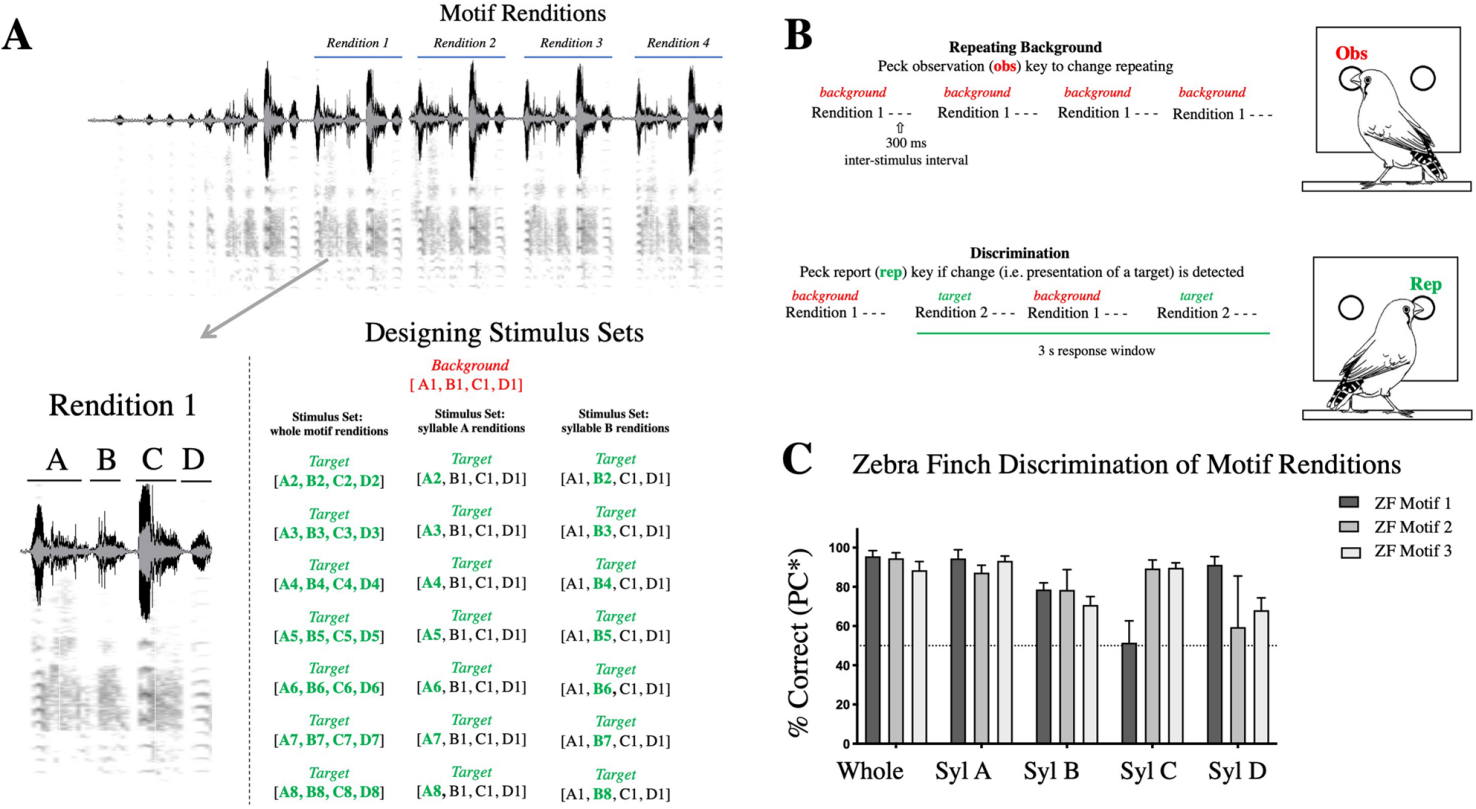
(**A**) Time waveform (top) and spectrogram (bottom) for a natural zebra finch song bout (made in Sonic Visualiser^51^). The blue lines denote distinct motif renditions, composed of the same four syllables (indicated as discrete notes via the time waveform). The panel below indicates how individual syllables were identified within a single motif rendition. A single motif (e.g. rendition 1) was used as a repeating background (ABCD, with fixed inter-syllable silences) and targets (sounds different from the background that birds were tasked with discriminating) consisted of different renditions of all syllables or one of the syllables. The right panel shows exemplar stimulus sets used to test discrimination ability among renditions of motifs and syllables. Birds were tested on five stimulus sets per male’s motif (three of which are shown), for each of three males. For the whole motif rendition set, the background was composed of each syllable from one motif rendition and each of the seven targets were comprised of each syllable from a different rendition (*stimulus set: whole motif renditions*). For the other four stimulus sets, the background was again composed of each syllable from a motif rendition, and each target contained a different rendition of a single syllable and the same rendition as the background for the other syllables (*stimulus set: syllable A renditions and syllable B renditions*). Target syllables differing from the background are green and bolded. (**B**) Schematic of the behavioral operant task. While listening to a repeating background sound, a bird can initiate a trial by pressing the observation key. After a 2–7 s random interval, another peck to the observation key resulted in the presentation of a target sound (a different rendition) alternated with the background sound. Each target is presented twice within a 3 s response window during which a peck on the report key is scored as a hit. (**C**) Average performance (corrected percent correct (PC*) for discriminating target stimuli, mean ± SEM, N = 4) on discrimination of motif and syllable renditions from three male zebra finch motifs (ZF Motif 1–3). The authors thank Shelby Lawson for the drawing of the zebra finch in (**B**).

Our first aim was to determine if zebra finches can discriminate motifs produced in the same context and from the same or adjacent song bouts. Figure 1A identifies distinct renditions of motifs and syllables within a single male’s song bout. Because every male’s song is quite different, we used song from three different males. We created five stimulus sets per male motif to be used in psychoacoustic experiments. Three examples of these five stimulus sets are shown in the bottom panel of Fig. 1A. These are composed of near-identical renditions of whole motifs and motifs where a single syllable was replaced with one from a different rendition. The acoustic similarity between renditions was assessed using the percent similarity score in Sound Analysis Pro 2011 (SAP), which uses feature-based metrics of Euclidean distances^50^. By this measure, renditions of whole motifs were between 91 and 99% similar.

The discrimination ability of four zebra finches (two males and two females) was tested using an operant psychoacoustic paradigm (Fig. 1B). Overall, zebra finches were easily able to discriminate all motif and syllable renditions for each of the three male’s motifs (ZF Motif 1–3) (Fig. 1C). While performance was quite good overall, individual performance was statistically different for tests on syllable renditions, depending on motif and syllable (lmer model: motif * syllable interaction, χ^2^ = 16.20, df = 6, p = 0.013)—this is particularly evident when looking at performance on syllables C and D (Fig. 1C). This raises the question of why certain syllables may be more discriminable than others.

### Zebra finch performance is influenced by syllable features

Zebra finch song syllables can be categorized into distinct syllable types (7–12 types) using acoustic features that are standard in many sound analysis programs^49,52,53^. In order to investigate how performance was related to syllable features, we quantified the following acoustic measures for each syllable from all three male’s motifs used as background stimuli: duration, fundamental frequency, mean frequency, peak frequency, goodness of pitch (referred to here as “harmonicity”), frequency modulation, amplitude modulation, entropy, spectral continuity over time, and spectral continuity over frequency.

**Figure 2:**
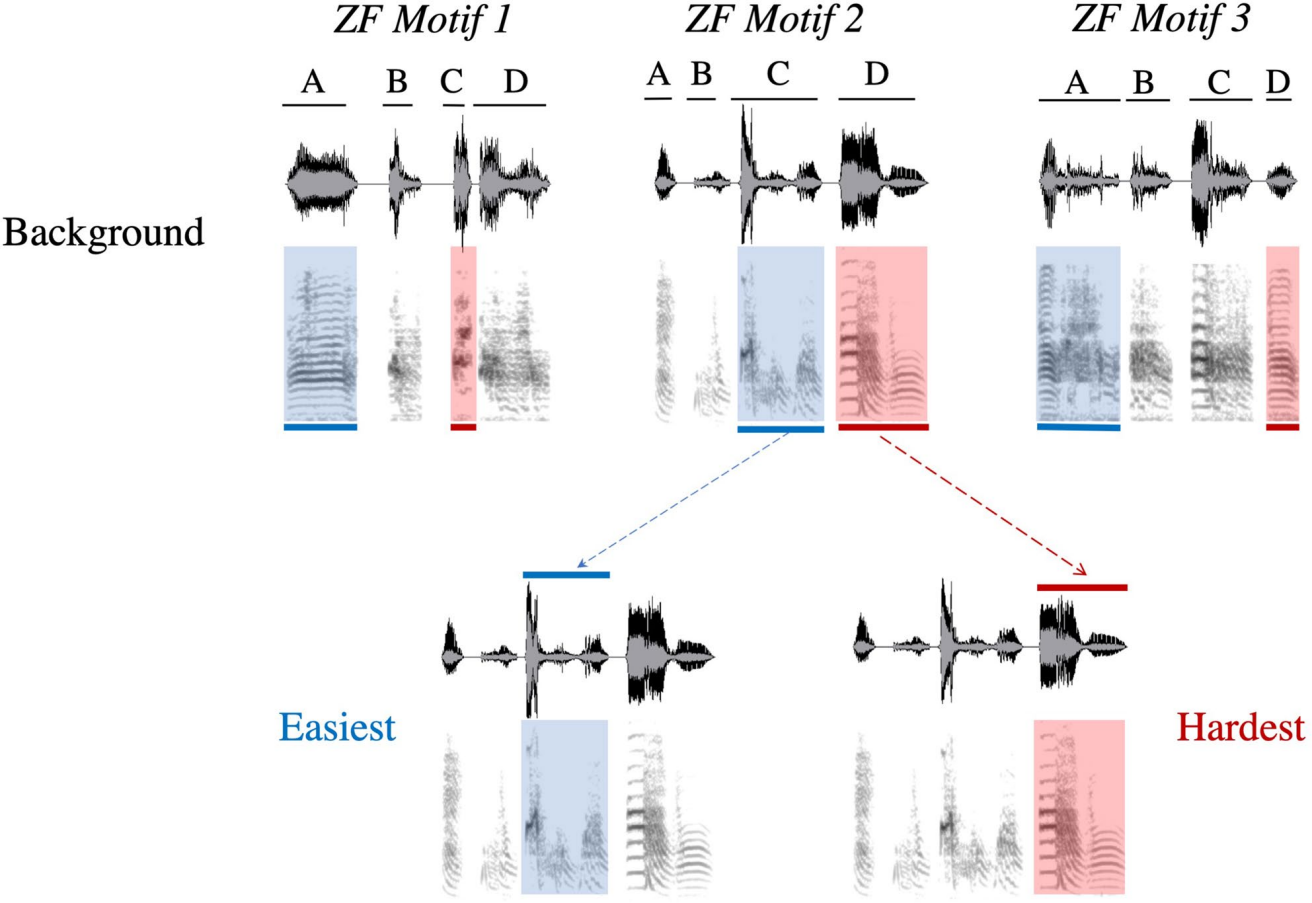
Time waveform (top) and spectrogram (bottom) for each background stimulus used for each of the three male’s motifs used in this experiment (made in Sonic Visualiser^51^). For each motif, the syllables are indicated (**A**–**D**) based on their position in the motif. For each motif, the syllable rendition that was easiest across birds is highlighted in blue and the syllable that is the hardest is indicated in red. For ZF Motif 2, the spectrograms and time waveforms for the easiest and hardest syllable rendition targets are depicted below the background stimulus. Note that while the spectrogram and time waveform are useful for visualizing the acoustic differences between types of syllables, they do not capture the rendition-to-rendition acoustic variation that zebra finches are so easily able to discriminate.

We used linear regression models to ask what features of the background syllables explained differences in performance on discriminating syllable renditions (Table 1). Overall, performance was related to mean frequency, duration, and harmonicity of the background syllables, such that higher frequency syllables tended to be more easily discriminated and, depending on the motif, syllables longer in duration and more harmonic were more easily discriminated. Figure 2 shows which syllable renditions were easiest and hardest for each motif. Note that while the spectrogram and time waveform are useful for visualizing the acoustic differences between types of syllables, they do not capture the rendition-to-rendition acoustic variation that zebra finches are so easily able to discriminate. Still, these results provide insight into what types of syllables might be particularly good sources for behaviorally relevant information in rendition-to-rendition variation.

**Table 1:** Results of linear regression models asking what types of syllables were most easily discriminated.

What features of syllables explain performance differences in syllable rendition discrimination?			
	χ^2^	Adj P-value	R^2^
**Fixed effect**			
FundFreq	3.77	0.224	0.152
MeanFreq	9.27	0.023*	0.294
PeakFreq	4.13	0.224	0.170
FreqMod	1.40	0.630	0.091
AmpMod	2.95	0.322	0.192
Entropy	1.65	0.598	0.101
Contt	0.03	0.993	0.032
Contf	0.67	0.993	0.175
**Interaction**			
Duration:Motif	12.81	0.023*	0.262
Harmonicity:Motif	14.56	0.021*	0.265

Each model consisted of mean PC* scores on each task other than the whole motif renditions (4 syllable rendition tasks for each of the 3 motifs) for each subject as the response variable (37 observations), the fixed effects were an acoustic feature of the relevant background syllable (e.g. duration), motif set (3 levels), and the interaction between motif set and acoustic feature, and the random effects were subject and task (12 levels) to account for the repeated measures design of the experiment. The formulas for the models were as follows: PC* ~ Feature*Motif + (1|Subject) + (1|Task). Chi-square (χ^2^) values (comparing the full model against a model that includes all other terms) are given for the fixed effects involving an acoustic feature, unless the interaction between acoustic feature and motif set was significant, in which case that is reported. P-values for chi-square tests were adjusted using the Benjamini–Hochberg false discovery rate procedure. Marginal R^2^ is given for the fixed effects for each model. * < 0.05.

*FundFreq* fundamental frequency, *MeanFreq* mean frequency, *PeakFreq* peak frequency, *FreqMod* frequency modulation, *AmpMod* amplitude modulation, *Contt* spectral continuity over time, *Contf* spectral continuity over frequency.

### Zebra finches can also discriminate natural variation in human vowels

**Figure 3:**
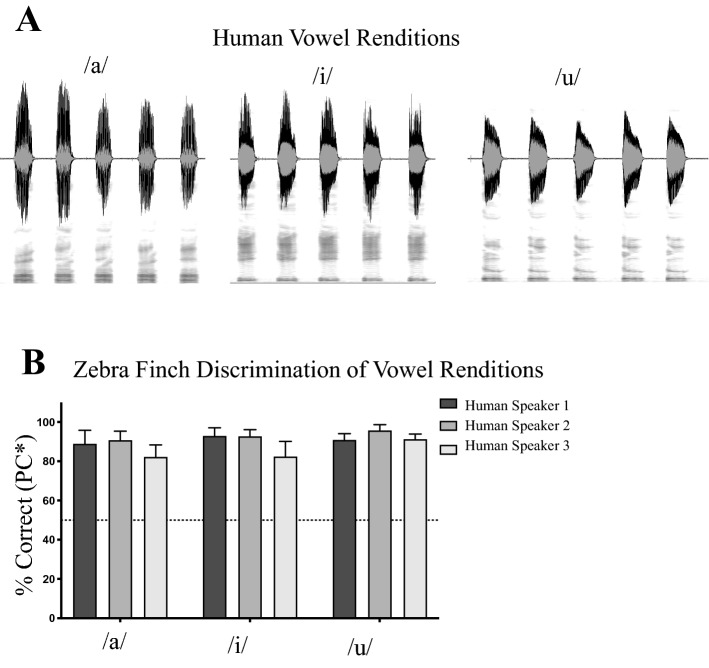
(**A**) Time waveform (top) and spectrogram (bottom) of a single human speaker producing the sustained vowels: /a/ /i/ /u/, as in “father”, “eat”, and “goose” respectively (made in Sonic Visualiser^51^). To prepare vowels for psychoacoustic tests, the middle section of a sustained vowel was extracted and given a 5 ms cosine rise/fall time. (**B**) Performance (PC*, mean ± SEM, N = 3) on discriminating the different renditions of extracted vowels from three human speakers.

As a test of whether zebra finch discrimination of natural variation is specific to their own vocalizations or reflects a more general capacity, we also tested the birds on renditions of natural human vowels (/a/, /i/, and /u/) (Fig. 3A). Just as with the conspecific vocal stimuli, zebra finches easily discriminated between renditions of spoken human vowels, showing that this perceptual sensitivity to acoustic details is not specific to conspecific signals (Fig. 3B).

### Human discrimination of natural acoustic variation

**Figure 4:**
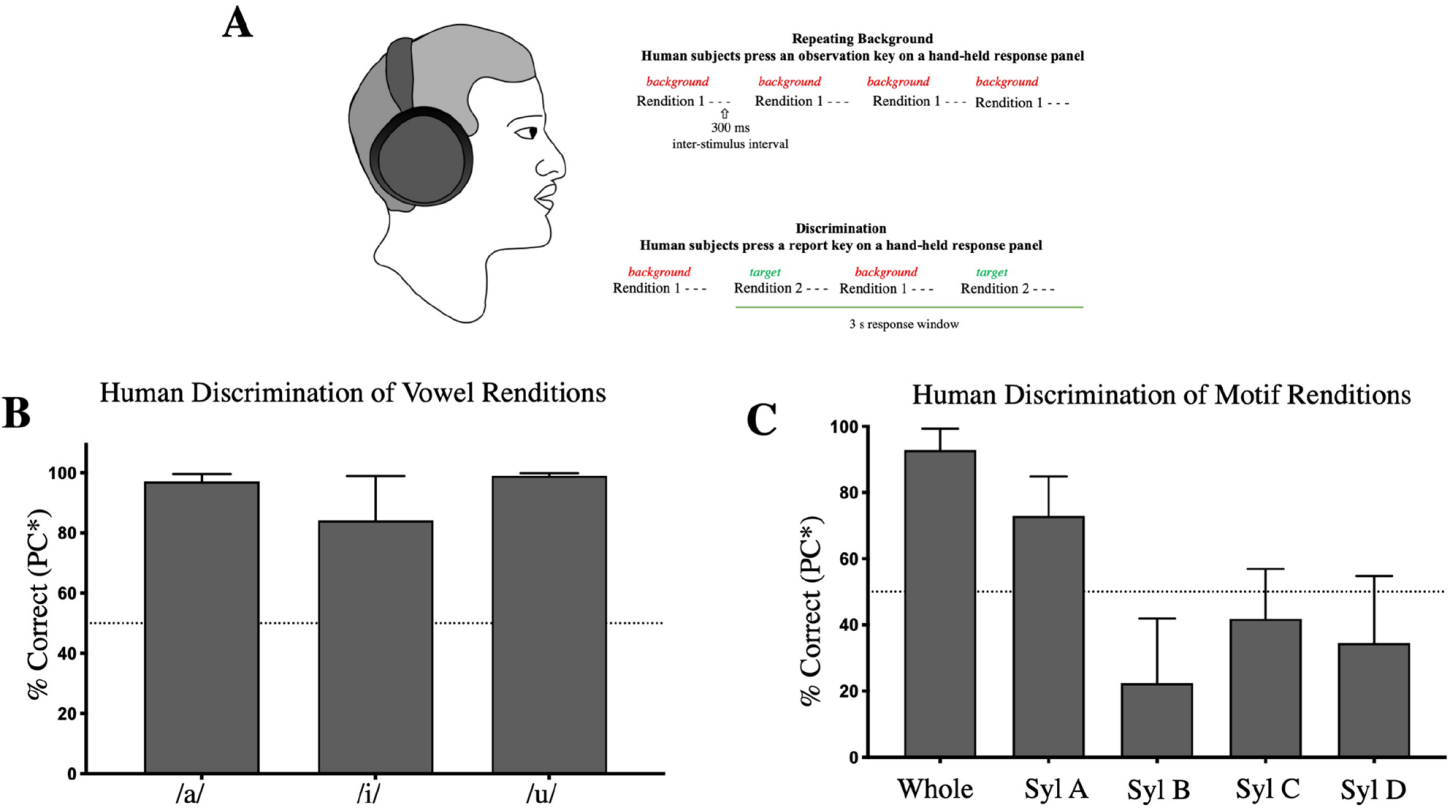
(**A**) Description of human psychophysical testing. Subjects were stationed at the same computers which controlled the bird operant tasks, outfitted with Sennheiser HD280PRO headphones, and given a response panel with two keys. Subjects were told they would be listening to a repeating background sound, during which they should press the observation key to cause a change in the background and to press the report key whenever they heard a change. (**B**) Performance (PC*, mean ± SEM, N = 3) on discriminating the different renditions of vowels from a single human speaker. (**C**) Performance (PC*, mean ± SEM, N = 3) on discriminating the different renditions of zebra finch song.

For comparison, we also tested three human participants on the different rendition stimulus sets for one male’s motif (ZF Motif 2) and one speaker’s vowels (Fig. 4A). Human participants easily discriminated among renditions of spoken vowels (Fig. 4B). The ability of human participants to discriminate among renditions of song motifs and syllables was more mixed. Humans performed very well at discriminating whole motif renditions and syllable A; however, their mean performance was below 50% PC* for the other syllables (Fig. 4C).

## Discussion

Perhaps because the linguistic content (phonology, syntax, semantics) of human speech is so unique, much of the research comparing acoustic communication systems in humans and non-human animals has focused on finding parallels to these components of human language. However, the non-verbal acoustic features of vocalizations are also rich sources of socially-relevant information across taxa. Here, we show that zebra finches are easily able to discriminate renditions of syllables and motifs of multiple male’s songs. Furthermore, zebra finches are also easily able to discriminate between renditions of human vowels from multiple speakers. Additionally, we show that human subjects perform very well in discriminating motif and vowel renditions using the same behavioral paradigm. These results support the notion that the perception of subtle acoustic variation in the utterances of vocal signals is a fundamental aspect of acoustic communication across species.

A historical reliance on spectrographic analysis of birdsong based on visual representation of song in sonograms has biased observers away from the potential importance of subtle acoustic details like fine structure, which are not easily discerned in sonograms^54^. Indeed, the acoustic variation that humans and birds discriminate here are not evident in the spectrogram or time waveform. Instead, researchers have often assumed that important information is contained in the sequential patterns of birdsong (perhaps reflecting an assumption of linguistic content). However, experiments both in the field and lab show that sequence may not matter much, at least in some species^16,48^. By contrast, previous work has shown that birds, compared to humans and other mammals, have superior auditory temporal resolution and excel in the ability to discriminate changes in acoustic fine structure, or rapid fluctuations in frequency and amplitude, of both synthetic and natural complex signals^46,55–59^. For zebra finches particularly, there is evidence that some of the smallest differences in acoustic fine structure found in their vocal signals may encode information about sex, call type, and individual identity^47^. This exquisite sensitivity to syllable details contrasts with the birds’ relative insensitivity to changes in syllable sequence in a motif^27,48^.

In the current experiment employing natural complex stimuli, we cannot determine whether the birds attended more to the faster changes in amplitude and frequency associated with fine structure or slower envelope cues. However, based on the abilities described in the previous paragraph, we might predict that zebra finches can discriminate across renditions of song syllables and human vowels based on variation in acoustic fine structure alone. Here, we used traditional acoustic measures in SAP to explain variation in performance across motifs and syllables. While these measures most certainly do not capture all the relevant acoustic features of the syllables, we provide evidence that syllables higher in mean frequency and, depending on the motif, syllables longer in duration and more harmonic may be particularly rich in behaviorally relevant information. In order to identify more precisely the perceptual mechanisms underlying the discrimination of natural renditions, further research using a wider set of stimuli, including experimentally manipulated sounds, would be needed to disentangle envelope and acoustic fine structure cues.

Regardless of the perceptual mechanisms, here we provide strong evidence that zebra finches can perceive some of the smallest acoustic variation present in their song. The more challenging question is whether and to what extent this variation is behaviorally relevant across social contexts. Certainly, there are already several lines of evidence that fine-grained variation in the acoustic structure of zebra finch calls conveys significant information (i.e. motivational state and breeding condition^32,60–62^), and this may be true for song as well^27,44^. Furthermore, zebra finch song is used for courtship and pair maintenance, so rendition variability could convey properties of the sender such as mate quality, hormonal condition, and motivational state. Zebra finch song is also modulated by social context and can be classified as “directed” (female-directed) or “undirected”. The acoustic differences between these contexts has been shown to be important for mate choice^63^. Both directed (which we use in this study, see “Methods” section) and undirected song are composed of the same stereotyped motif but directed song is faster, longer, contains more introductory notes and has increased stereotypy at the level of the syllable, motif, and whole song^63^. Combined, these lines of research highlight the potential importance of these subtle acoustic features, including acoustic fine structure, for communication. Specific manipulation of acoustic fine structure in studies of natural behavior would be pivotal in testing the idea that it is a primary carrier of information in song.

Human participants in our study easily discriminated variation in human vowels and some of the song syllables. While humans are exquisitely primed to extract linguistic content^64^, a long line of research clearly shows that they are also sensitive to paralinguistic acoustic features of the voice in both speech and non-speech vocalizations^37,65^. In a recent study, Spierings and ten Cate (2014) tested zebra finches and humans on categorizing speech sounds based on prosodic (pitch, duration, and amplitude) or sequence (xxyy vs xyxy) cues^66^. When responding to test stimuli where subjects could use both cues, zebra finches always used prosodic cues more than sequence cues, while human participants used both. Thus, there is strong converging evidence that zebra finch song perception primarily focuses on acoustic details akin to the paralinguistic features of human speech.

Our current study adds to a growing body of research illustrating the parallels between the non-verbal content of human speech and acoustic communication in animals. Even though zebra finch song seems to lack linguistic content, the emotion and meaning contained in the acoustic fine structure of song could very well exceed that of human speech. Our study focused primarily on birds, but subtle acoustic variation in the “voice,” within categories and units of vocal signals, is well-documented across a range of species, including anurans (reviewed in^67^), such as in the territorial calls of Central American tree frogs^68^, and numerous species of mammals and birds. Altogether, there are many lines of evidence which would suggest that acoustic communication of affective state is a shared, foundational property of vocal communication systems^19,65,69^.

## Methods

### Subjects

Adult male and female zebra finches (> 120 days old) were used for these experiments. For the psychophysical experiments, five zebra finches (three males and two females) in total were used. Three zebra finches (one male and two females) were tested on all 15 stimulus sets for the zebra finch motif and syllable rendition experiments. An additional male zebra finch was tested on two of the stimulus sets. Three zebra finches (one male and two females) were tested on all 9 stimulus sets for the vowel rendition experiments. Two of the birds (two females) were tested on both the zebra finch song and vowel experiments. During the experiments, subjects were housed in individual cages on a light cycle of 8L:16D. Birds were mildly food deprived at about 90–95% of their free feeding weight to ensure they were motivated to engage in the psychophysical testing. White hulled millet was used as a food reward in the testing apparatus and birds received an additional portion of pellet or mixed seed at the end of the day. Birds also had access to grit and, occasionally, vegetables, fruit, or hard-boiled egg. Additionally, three human subjects were tested using the same psychophysical paradigm.

### Preparation of zebra finch stimuli

We recorded directed song from three zebra finches in a foam-covered room. Recordings were made using a tie-clip microphone (AKG C417) and a Zoom F8 multitrack field recorder (sampling rate of 44.1 kHz). Songs were viewed in Adobe Audition (v. 2015.2), and motifs were selected that did not have competing background noise (i.e. wing fluffs, cage noises, and female calls, etc.) (Fig. 1). Using Adobe Audition, motifs were high-pass filtered with a cutoff frequency of 350 Hz. Consecutive motif renditions were taken when possible, on the assumption that this would maximize the similarity in acoustic fine structure of syllables. Eight renditions of individual syllables were then extracted from eight motif renditions for further preparation to be used as psychophysical stimuli in these experiments. The same eight motif renditions were used for each syllable type, and extracted syllables were given identifiers based on the syllable type (position in the motif A–D) and motif rendition (1–8). Thus, for three zebra finch songs, we had syllables A1–A8, B1–B8, etc.

After individual syllable renditions were extracted, motif stimuli were generated in MATLAB (MathWorks, Natick, MA). Stimulus motifs were created, making two adjustments in order for the stimuli to be appropriate for psychophysical testing. First, inter-syllable silences were fixed for each stimulus motif so that birds could not use differences in inter-syllable silences as a cue. These inter-syllable silences were based on the naturally occurring silences for a single rendition of that male’s motif. Second, each extracted syllable was given a 5 ms cosine rise/fall time. Consistent rise/fall times are necessary to preserve the acoustic features of syllables following inter-syllable intervals of complete silence.

In the psychophysical discrimination experiments, the repeating background stimulus was a motif with syllables in the natural order. For the whole motif rendition set, the background was composed of each syllable from one motif rendition (A1, B1, C1, D1) and each of the seven targets were comprised of each syllable type from a different rendition (e.g. A2, B2, C2, D2). For the other four stimulus sets, the background was again composed of each syllable from one motif rendition (A1, B1, C1, D1), and for each of the seven targets a single syllable was substituted from a different rendition (e.g. A2, B1, C1, D1; A3, B1, C1, D1) (Fig. 1).

### Description of acoustic features

We analyzed the acoustic features of the motif renditions using Sound Analysis Pro 2011 (SAP)^50^. We quantified the acoustic similarity between each target rendition and the background using the percent similarity score in SAP, which uses feature-based metrics of Euclidean distances. In addition, we used SAP to describe key features of syllables (i.e. duration, fundamental frequency, mean frequency, peak frequency, goodness of pitch, frequency modulation, amplitude modulation, entropy, spectral continuity over time, and spectral continuity over frequency) by using the feature statistics to generate averages of the above features from the onset to the offset of each syllable. Goodness of pitch (referred to as “harmonicity” throughout the manuscript) is an estimate of how periodic the sound is, and values are higher when sounds are more harmonic and less noisy. Entropy is based on Wiener entropy values and estimates the noisiness or randomness of the sound. Spectral continuity measures continuity of frequency contours across time windows (whether spectral slopes are continuous). Spectral continuity over time values are high when the contours are long and spectral continuity over frequency values are high when the frequency range of the contours is high.

### Preparation of vowel stimuli

Recordings were made of three human male speakers producing the sustained vowels /a/ /i/ /u/. Speakers were instructed to utter each instance of the vowel as consistently as possible. Recordings from one speaker were made in a foam-covered room by tie-clip microphones (AKG C417) and a Zoom F8 multitrack field recorder at a sampling rate of 44.1 kHz. Recordings from the other two speakers were made in an acoustically treated room by an AKG 414 ULS condenser mic into an apogee duet into Ableton Live at 44.1 kHz. A 150 ms section of the sustained vowel was extracted from the middle of each utterance in Adobe Audition and given a 5 ms cosine rise/fall time. Birds were additionally tested on two sets of stimuli created in the same way but with a 100 ms section of the vowel extracted from the middle of each utterance. A stimulus duration of 100 ms was chosen because this is similar to the average duration for zebra finch song syllables^53^.

### Apparatus

As described previously, e.g.^48,56,57^, birds were trained and tested in a wire cage (23 × 25 × 16 cm) anchored inside of a sound-attenuated chamber (Industrial Acoustics Company, Bronx, NY, model IAC-3) lined with acoustic foam. Two response keys, each consisting of an LED attached to a microswitch, were mounted to the wall of the cage directly in front of a perch. Millet was delivered through activation of a solenoid. Stimuli were stored as wav files on an Intel Core 2 Duo computer (Mid Atlantic Data Systems, Gaithersburg, MD), which controlled the psychoacoustic experiments. The computer operated a Tucker Davis Technologies (TDT) System 3 module (Alachua, FL) that sent signals through a Crown D-75 amplifier (Elkhart, IN) and to an Orb full range point source speaker (Model Mod 1, Orb Audio, Sherman Oaks, CA), which was placed 40 cm above the bird’s head when standing on the perch. All stimuli were resampled to 24,414 Hz as required for the TDT system.

### Psychophysical task

Birds were trained through operant conditioning to perform a psychophysical discrimination task. The training and testing procedures have been described in detail previously, e.g.^48,56,57^. Pure tones were used in training birds on the task and individuals were tested for months to years with this psychophysical task on a variety of stimuli. Subjects were not previously tested on the rendition stimuli used in these experiments. The discrimination task proceeded as follows: the birds listened to a repeating background sound and pecked the left LED (the observation key) to initiate a trial. This first peck on the left LED initiated a random interval of 2–7 s. Following this random interval of 2–7 s, another peck on the observation key resulted in the presentation of a target stimulus. If the bird pecked the right LED (the report key) within 3 s following the presentation of a target stimulus, this was considered a “hit” and they received a positive reinforcement (2 s access to millet from a food hopper) (Fig. 1B). Birds generally performed 100 trials in a session consisting of 10 × 10-trial blocks. Three of the trials within a 10-trial block were sham trials in which the background sound was inserted instead of a target, providing a means of assessing false alarm rate. If the bird pressed the report key during a sham trial (considered a “false alarm”) or outside of the response window, they received a mild punishment where the house lights were turned off for a short blackout period, which was set between 1–14 s at the start of the session depending on the response proclivities of each individual bird. If a bird performed with a high false alarm rate on a session, then the blackout time was set higher on the subsequent session. All stimuli were normalized to be played at 65 dBA measured with an SPL meter (BK Precision model 732) at approximately the location of the bird’s head when positioned in front of the observation key. Motifs were presented at a rate of 1/s so that there was always about a 300 ms interval between the end of one motif and the beginning of another. Thus, each bird had the opportunity to hear the target alternated with the background twice during the response window.

For each stimulus set, birds were tested in 100-trial sessions until their performance stabilized over 200 trials with a false alarm rate (# of sham trials resulting in a false alarm/total # of sham trials) below 20% for each 100-trial block and a difference in hit rate (# of target trials resulting in a hit/total # of target trials) less than 15% between blocks. It took birds 2–4 100-trial blocks to achieve stable performance. Overall, the average false alarm rates across birds was very low (mean ± SEM: 4.12 ± 2.32).

However, two separate individuals were not able to meet the false alarm criterion for one task (one bird on Motif 1 Syllable C Renditions and a different bird on Motif 2 Syllable D Renditions). In these two cases, the birds were tested on additional 100 trial blocks until their false alarm rate stabilized (28% in one case and 36% in the other). In these two instances, the birds also had low hit rates on those tasks (26% and 57%, respectively). As these birds met the false alarm criterion on all other tasks, the high false alarm rate was indicative of their difficulty in discriminating among renditions of those particular stimuli.

### Human testing

We also tested humans on the same stimuli used to test the birds and on a similar psychophysical procedure. Human subjects were recruited from staff and students in the lab and informed consent was obtained from all participants. Participants had no prior experiences with these stimuli. The human testing procedure was modeled after the procedure used with the birds. Subjects were stationed at the same computers which controlled the bird operant tasks, outfitted with Sennheiser HD280PRO headphones, and given a response panel with two keys. Subjects were told they would be listening to a repeating background sound, during which they should press the observation key to effect a change in the background and to press the report key whenever they heard a change. Subjects were tested on a subset of the stimulus sets: a single bird’s motif (motif 2) and a single speaker’s vowels (speaker 2). Humans ran for one 100-trial session on each task.

### Analysis

Performance (hits/misses/false alarms) on each task was summarized in 100-trial blocks for each individual and pooled together to calculate an averaged hit rate for each target and false alarm rate for each 100-trial block. The 200 trials that met the criterion were averaged and used for analysis. We used corrected percent correct (PC*) as a performance measure in order to minimize effects of different false alarm rates on each task^70,71^. Hit rates and false alarm rates were used to derive PC*:

$${\text{PC}}* \, = { 1}00 \times \left( {\left( {{\text{Hit rate }}{-}{\text{ False alarm rate}}} \right)/\left( {{1}00 - {\text{ False alarm rate}}} \right)} \right)$$

We conducted a linear mixed effects model using the function lmer from the lme4 package^72^ in R (v.3.6.3, R Foundation for Statistical computing)^73^ to test for differences in performance across motifs and syllables for each task other than the whole motif renditions (12 tasks total). In this model, PC* for each subject on each task was the response variable (37 observations), the fixed effects were motif (3 levels), syllable (4 levels), and the interaction between motif and syllable, and the random effect was subject. The formula for the model was as follows: PC* ~ Motif*Syllable + (1|Subject). We also used linear mixed effects models to ask what features of background syllables explained differences in performance. Each model consisted of PC* for each subject on each task (other than the whole motif renditions) as the response variable (37 observations), the fixed effects were an acoustic feature (e.g. duration) of the relevant background syllable, motif set (3 levels), and the interaction between motif set and acoustic feature, and the random effects were subject and task (12 levels). The formulas for the models were as follows: PC* ~ Feature*Motif + (1|Subject) + (1|Task). The function r.squaredGLMM from the MuMIn package^74^ was used to calculate marginal R^2^ for the fixed effects for each model. The function Anova from the car package^75^ was used to perform type 2 Wald chi-square tests, providing a chi-square (χ^2^) value and p-value for the fixed effects involving an acoustic feature in each regression model (comparing the full model against a model that includes all other terms). Adjusted p-values to account for multiple testing were calculated using the Benjamini–Hochberg false discovery rate procedure.

### Ethics

Animal procedures were approved by the University of Maryland Animal Care and Use Committee (protocol number: 1191420). These procedures followed the Animal Behavior Society (ABS) and Acoustical Society of America (ASA) guidelines for the use of animals in research. The procedures for human participant work was approved by the University of Maryland Institutional Review Board (protocol number: 1361480). These procedures followed the ASA guidelines for the use of human participants in research and informed consent was obtained from all participants.

## Data Availability

Data will be made available upon reasonable request.
